# Palliative Radiotherapy to Preserve Eyesight in a Recurrent, Difficult-to-Treat Basal Cell Carcinoma

**DOI:** 10.7759/cureus.77743

**Published:** 2025-01-20

**Authors:** Andra Ioana Copilău, Beatrice Bălăceanu-Gurău, Andrei Anghel, Irina Tudose, Mara Mădălina Mihai

**Affiliations:** 1 Dermatology Department, Elias Emergency University Hospital, Bucharest, ROU; 2 Oncologic Dermatology Department, Elias Emergency University Hospital, Carol Davila University of Medicine and Pharmacy, Bucharest, ROU; 3 Radiotherapy Department, Elias Emergency University Hospital, Bucharest, ROU; 4 Pathology Department, Elias Emergency University Hospital, Bucharest, ROU

**Keywords:** basal cell carcinoma of scalp, hedgehog pathway inhibitors, infiltrative basal cell carcinoma, periocular cutaneous malignancy, radiotherapy (rt), recurrent basal cell carcinoma, skin oncology, volumetric‐modulated arc therapy (vmat)

## Abstract

This case discusses an 85-year-old patient with a history of cataract causing severe right eye vision impairment and repeated surgeries for a basal cell carcinoma (BCC) on the right temple and its local recurrences (wide local excision in 2010; re-excision and reconstruction with a skin graft in 2017), who presented with progressive growth and extension of the skin tumour. Upon examination, an irregular, erythematous plaque with multiple ulcerations on the surface (the largest measuring 4 × 3 cm on the left temple) was observed. The lesion extended from one temple to the other, over the forehead, along the margins of the surgical skin graft, and invaded the upper left eyelid, with a protruding mass extending out of the orbit. A punch skin biopsy of the largest ulceration revealed basal cell carcinoma, infiltrative subtype, with areas of bone invasion. Given the difficult clinical scenario, the complex anatomical location, and the potential morbidity associated with surgery, the patient was deemed suitable for radiotherapy after a thorough evaluation. The patient showed good tolerance to treatment, with mild radiodermatitis managed topically, and achieved a satisfactory therapeutic response. Clinical and radiological evaluations demonstrated substantial regression in lesion size, no significant toxicities, and preservation of vision in the left eye. This case highlights the successful use of palliative radiotherapy in a patient with recurrent giant basal cell carcinoma of the upper face with orbital invasion, achieving eyesight preservation when surgery or systemic therapy were not viable options. Radiotherapy is emerging as a valuable treatment option for recurrent basal cell carcinoma in challenging anatomical locations. However, careful monitoring and rigorous treatment planning are essential forachieving favourable outcomes while minimizing side effects.

## Introduction

Basal cell carcinoma (BCC) is the most common cancer in humans, originating from the stem cells of hair follicles. Because cumulative ultraviolet (UV) exposure is the most important carcinogen, it typically develops on the sun-exposed skin of elderly people, most commonly located on the head and neck. Other causal or predisposing factors include environmental exposure to ionising radiation, arsenic, pigmentary features (fair, light phototypes), immunodeficiency, and genetic variants and mutations (MC1R gene variants, PTCH1, PTCH2, SMO, SUFU, and many more) [1, 2].

BCC presents with various clinical and histological features, ranging from superficial, nodular, micronodular, morpheaform, infiltrative, fibroepithelial, or basosquamous tumours. It may even show a combination of histological characteristics of different subtypes, each associated with distinct management and prognosis. While it is classified as an amelanotic skin tumour, some lesions can be pigmented. Upon clinical examination, BCC often manifests as a slow-growing, skin-coloured nodule with a pearly, glossy appearance and arborising vessels visible on the tumour surface; larger tumours may exhibit core ulceration [1]. Another common subtype is superficial BCC (sBCC), which is mostly associated with sporadic sun exposure and is more frequently found on the trunk of younger adults. Clinically, it appears as flat, erythematous, and scaly lesions with well-demarcated edges [2]. Sclerosing, desmoplastic, or infiltrative alterations are seen in fewer than 10% of BCCs. These lesions exhibit aggressive biological behaviour with subclinical extension and local destruction, along with higher rates of local recurrence, making them far more challenging to treat. Clinically, morpheaform (sclerosing) BCCs appear as infiltrating plaques with a glossy surface and ill-defined borders, mimicking scars or morphea plaques on the head or neck. Infiltrative BCCs clinically manifest as ill-defined, indurated, flat or depressed plaques that are white, yellow, or pale pink in colour. Papules, ulcerations, erosions, and crusts may also be present on the surface [3].

Giant BCCs represent a rare subgroup with a diameter greater than 5 cm, independent of histological subtype, local invasion, or metastasis, although each of these has been reported as a potential characteristic of giant BCC [4].

A BCC's natural history is often that of a slow-growing, non-aggressive skin cancer that begins as a tiny lesion and grows over years into a nodule or plaque, occasionally ulcerating, thus allowing time for proper diagnosis and treatment [1]. Recurrence and local destruction are the main complications following BCC surgical excision. The tumour's location (facial "H" zone), histological subtype, perineural invasion, immunosuppression, and previous recurrences all influence the risk of recurrence [2]. Although fewer than 1% of individuals will develop metastases, if left untreated, poorly managed, or unnoticed, BCCs can grow into large, locally advanced, and frequently deeply infiltrating tumours [1]. Invasive and/or neglected BCCs of the face show a higher risk of recurrence after treatment and may result in extensive skin, soft tissue, and bone destruction, leading to severe disfigurement, blindness, or even death.

The therapeutic approach includes standard surgical excision, Mohs micrographic surgery, curettage and electrodesiccation, cryosurgery, topical treatment, photodynamic therapy, or radiation therapy [1, 3]. In difficult-to-treat or locally advanced BCCs (laBCCs), systemic therapy with Hedgehog pathway inhibitors (vismodegib, sonidegib) or immunotherapy using anti-PD1 immune checkpoint inhibitors (cemiplimab) can provide viable alternatives. However, their high cost and lack of reimbursement may limit access to these therapeutic options [1, 3].

Whether curative or palliative, radiation therapy may yield impressive response rates and significantly improve the patient's quality of life. In the last few years, palliative radiotherapy has played an increasing role in managing laBCC that is inoperable or resistant to conventional treatment. The choice of therapy should depend on the tumour's size, the extent of surrounding structure involvement, the affected areas, the course of disease, and the patient's medical history [1, 3].

The primary objective of this case report is to demonstrate the efficacy of palliative radiotherapy in a complicated, difficult-to-treat, and recurrent case of BCC in a patient who declined surgery and systemic therapy.

This article was previously presented as a conference poster at the 33rd European Academy of Dermatology and Venereology Congress, Amsterdam, September 25-28, 2024.

## Case presentation

We report the case of an 85-year-old female patient with a history of cataract causing severe vision impairment in the right eye and repeated surgeries for a BCC of the right temple with multiple local recurrences (wide local excision in 2010; re-excision and reconstruction with a skin graft in 2017). She reported the progressive growth and extension of the skin tumour over the forehead and left temple, associated with pain and impaired vision, including an inability to fully open her left eye.

**Figure 1:**
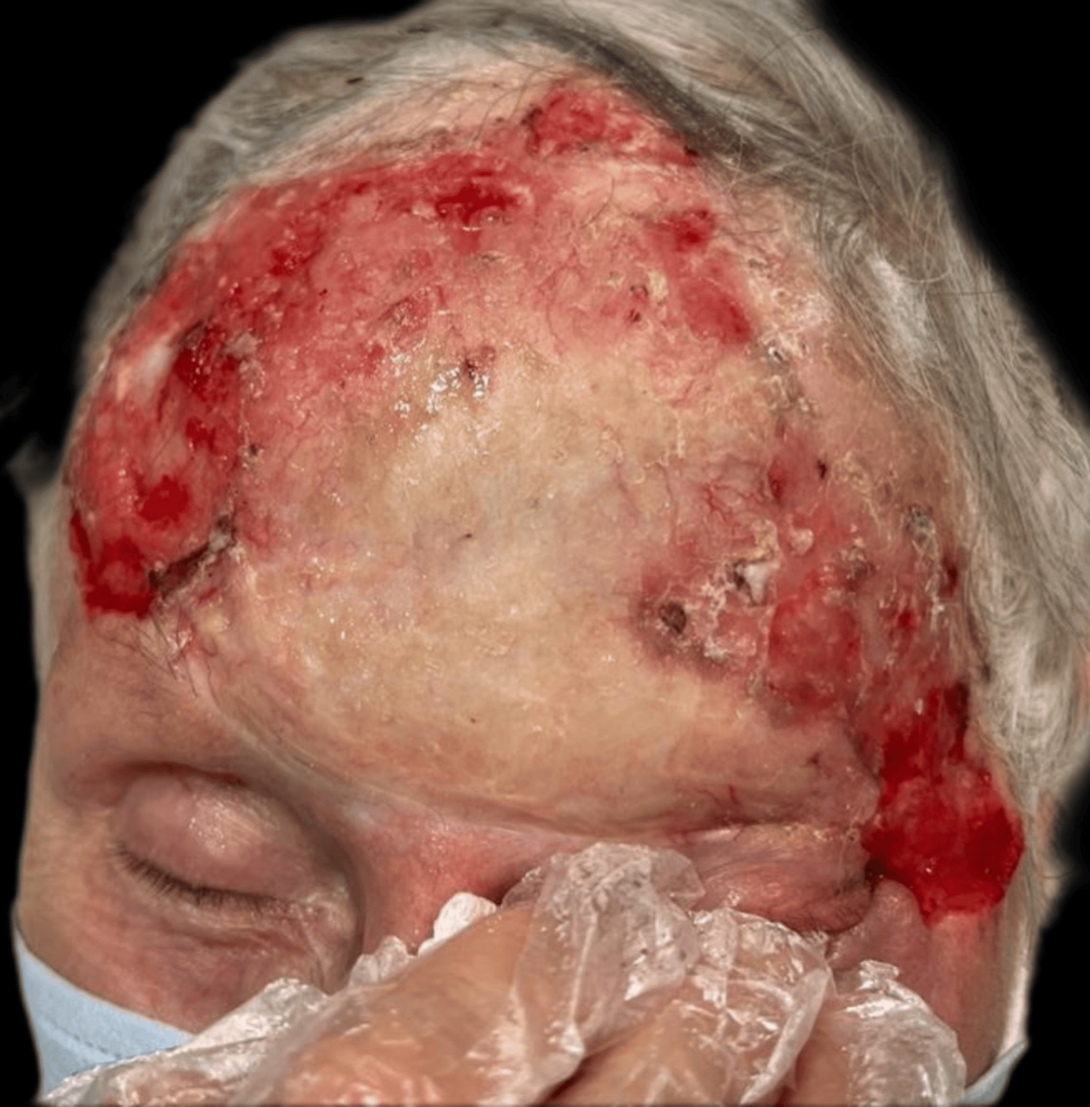
First clinical presentation: an irregular, erythematous large plaque with multiple ulcerations on the surface (the largest measuring 4 × 3 cm on the left temple), extending from one temple to the other, over the forehead, and along the margins of the surgical skin graft.

**Figure 2:**
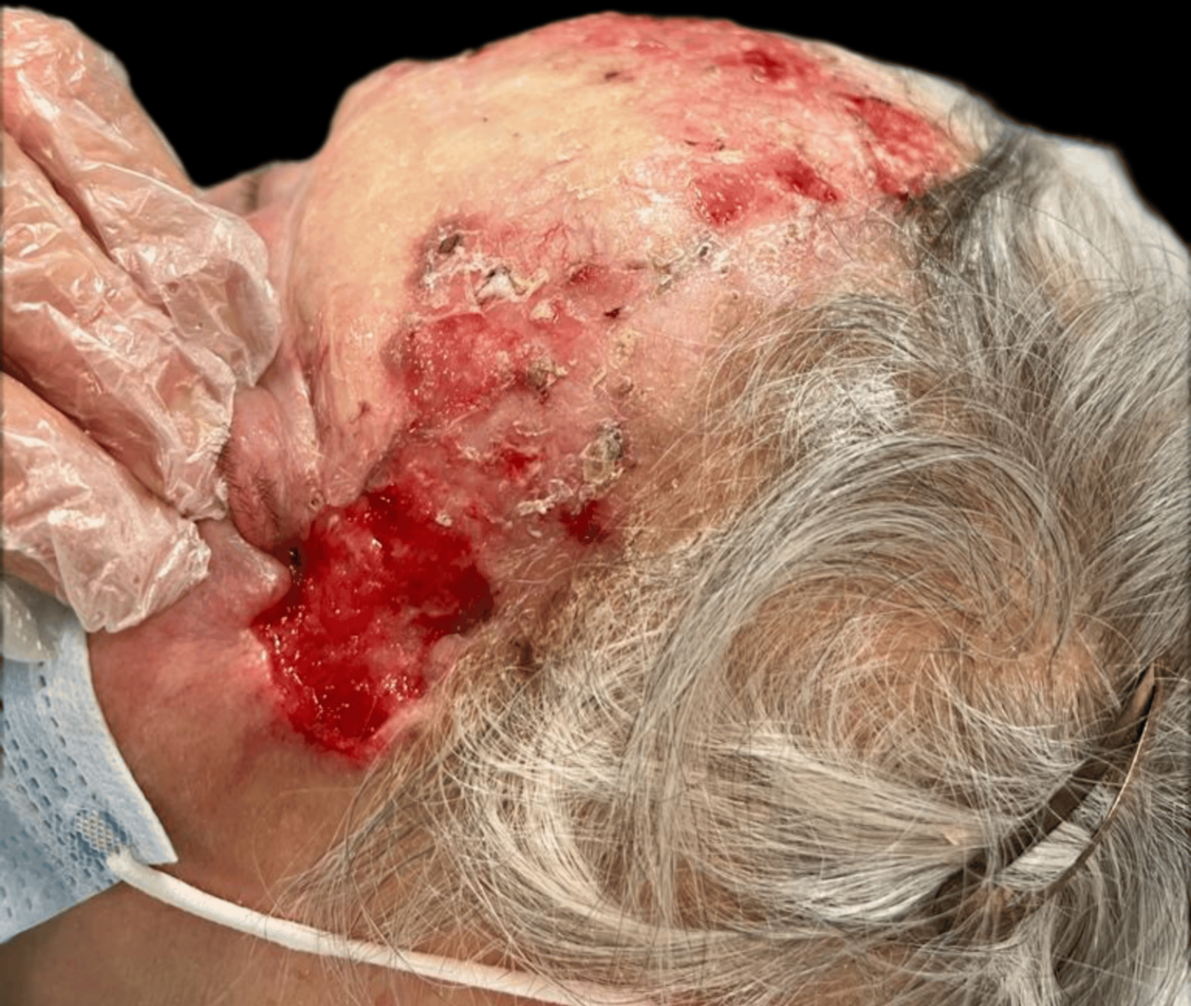
First clinical presentation: large superficial ulceration on the left temple and invasion of the lateral cantus.

**Figure 3:**
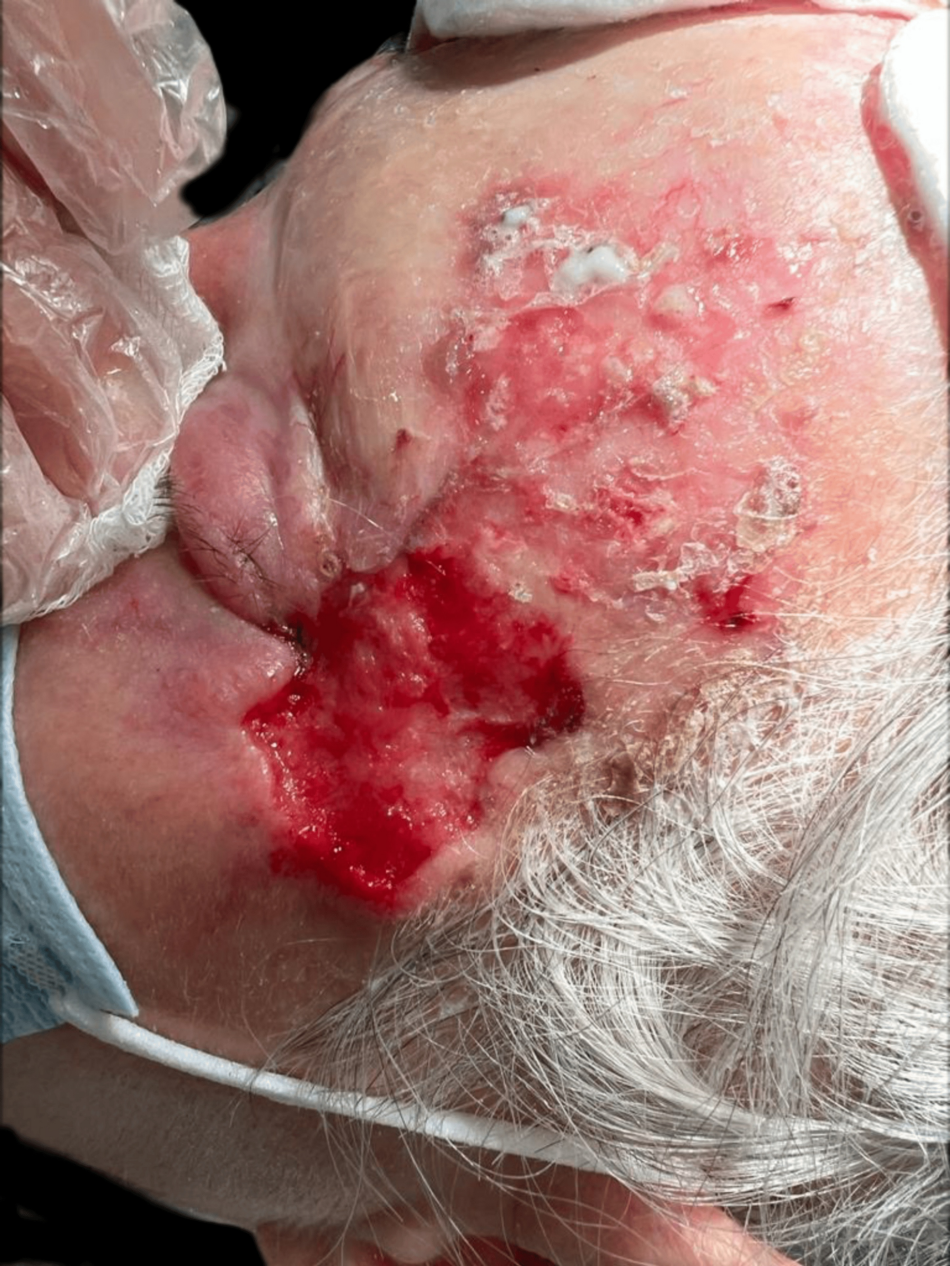
First clinical presentation: large superficial ulceration on the left temple on a scaling erythematous plaque and invasion of the upper left eyelid through the lateral cantus.

Upon examination, an irregular, erythematous large plaque was observed, with multiple ulcerations on the surface (the largest measuring 4 × 3 cm on the left temple). The lesion extended from one temple to the other, over the forehead, along the margins of the surgical skin graft, and involved the upper left eyelid with a protruding mass extending out of the orbit (Figures 1-3).

**Figure 4:**
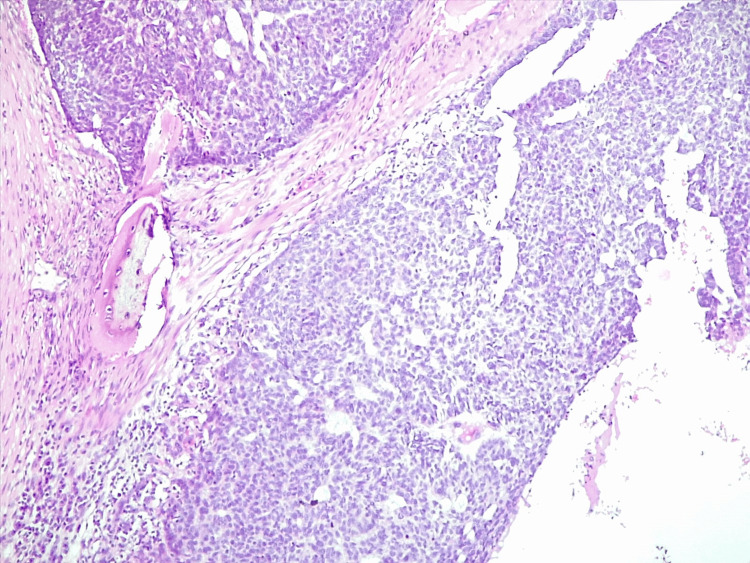
Section (H&E stain, x100) suggesting a diagnosis of basal cell carcinoma, infiltrative histological subtype, invading the bone structure.

**Figure 5:**
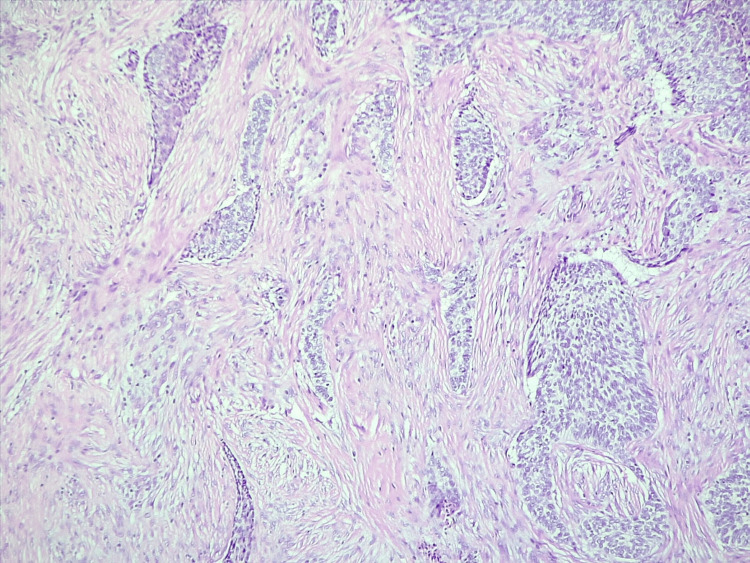
Section (H&E stain, x100) suggesting a diagnosis of basal cell carcinoma, infiltrative histological subtype.

A punch skin biopsy of the largest ulceration was performed, with results concluding the diagnosis of basal cell carcinoma, infiltrative subtype, with areas of bone invasion (Figures 4, 5).

**Figure 6:**
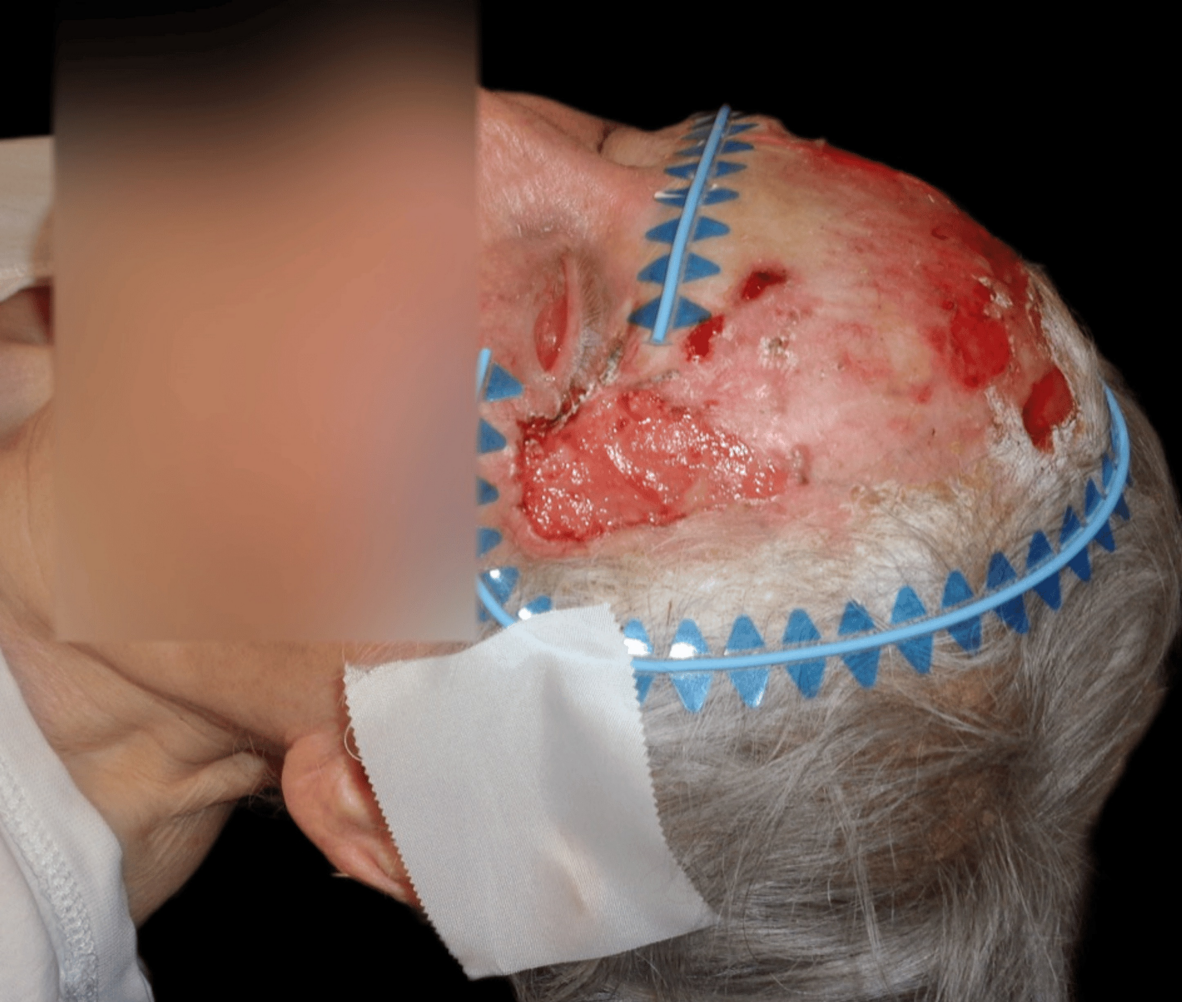
Lateral perspective of the target area prior to the first radiotherapy session.

**Figure 7:**
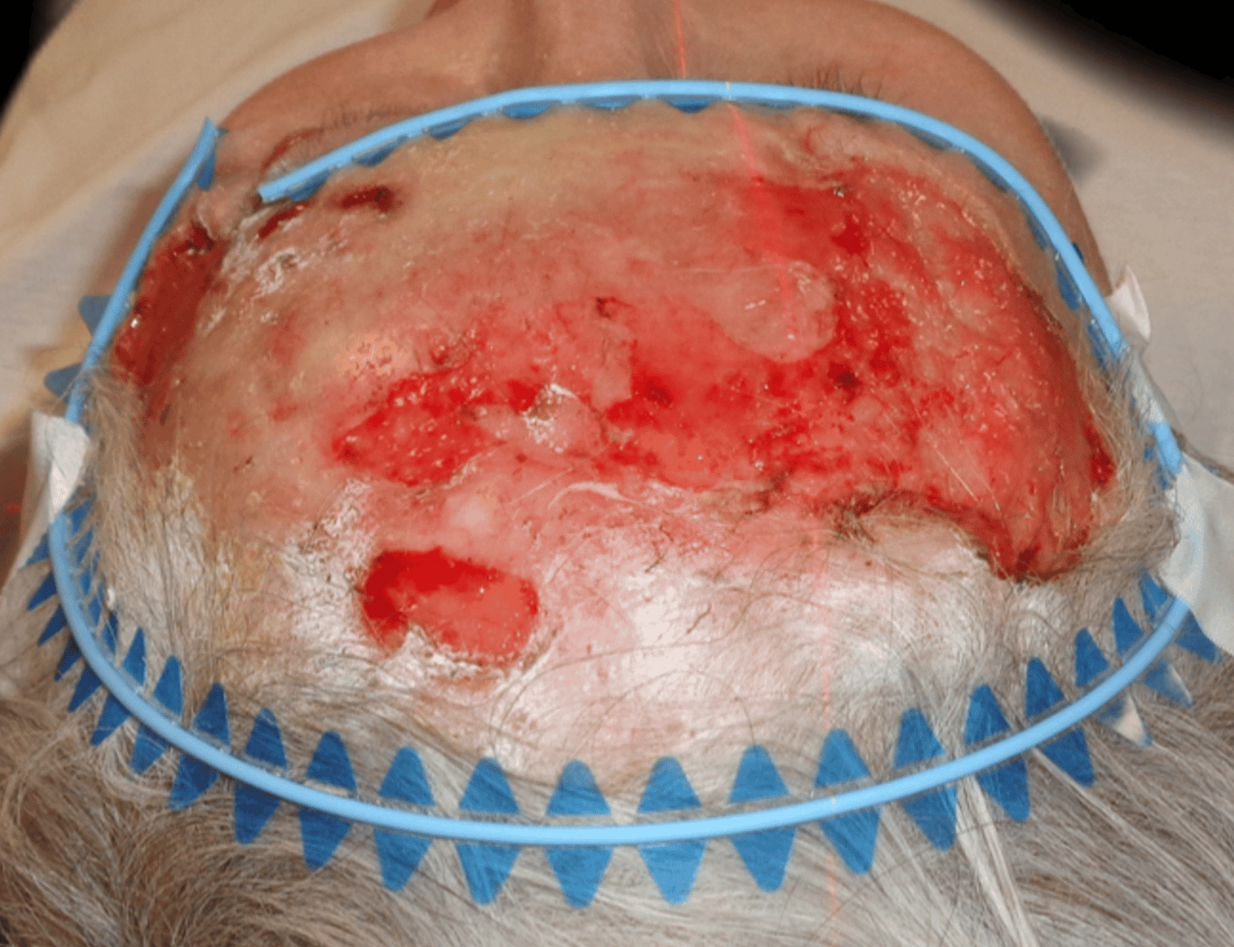
Cranial perspective of the target area prior to the first radiotherapy session

**Figure 8:**
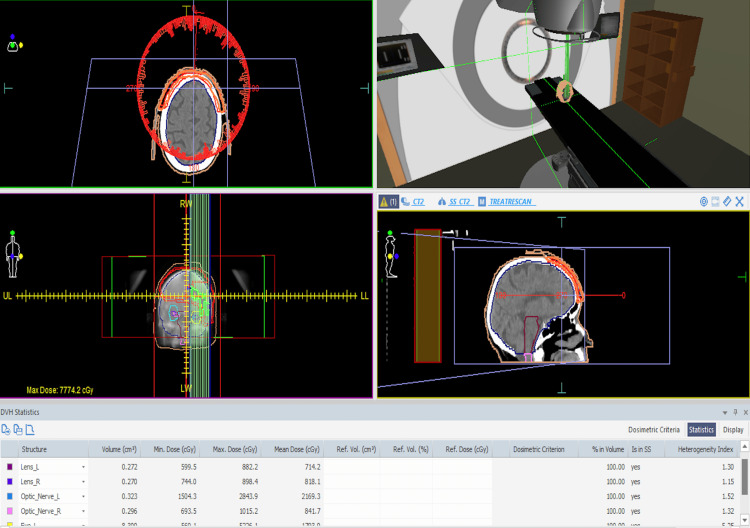
The irradiation protocol included a CT-based simulation to delineate the target area while reducing radiation exposure to adjacent sensitive structures.

**Figure 9:**
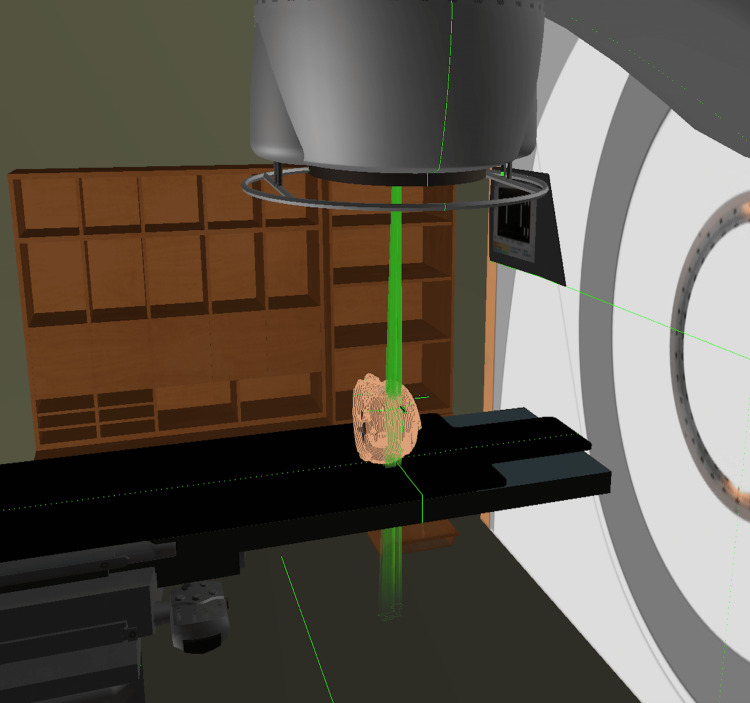
Simulation of the irradiation room and the position of the patient.

Given the difficult clinical scenario, the complex anatomical location and extension, and the potential morbidity associated with surgery, the patient was considered suitable for radiotherapy after a thorough evaluation of the case. The patient declined the surgical approach, as well as the radiotherapy procedure for the left orbit, in order to preserve eyesight since vision in the right eye was already severely impaired. Systemic therapy with Hedgehog inhibitors was not feasible because the treatment cost was not covered by the National Health Insurance Company in our country, and the long-term financial burden was too great. The radiotherapy protocol involved CT-based simulation to precisely delineate the target area while minimising radiation exposure to adjacent sensitive structures (Figures 6-9).

**Figure 10:**
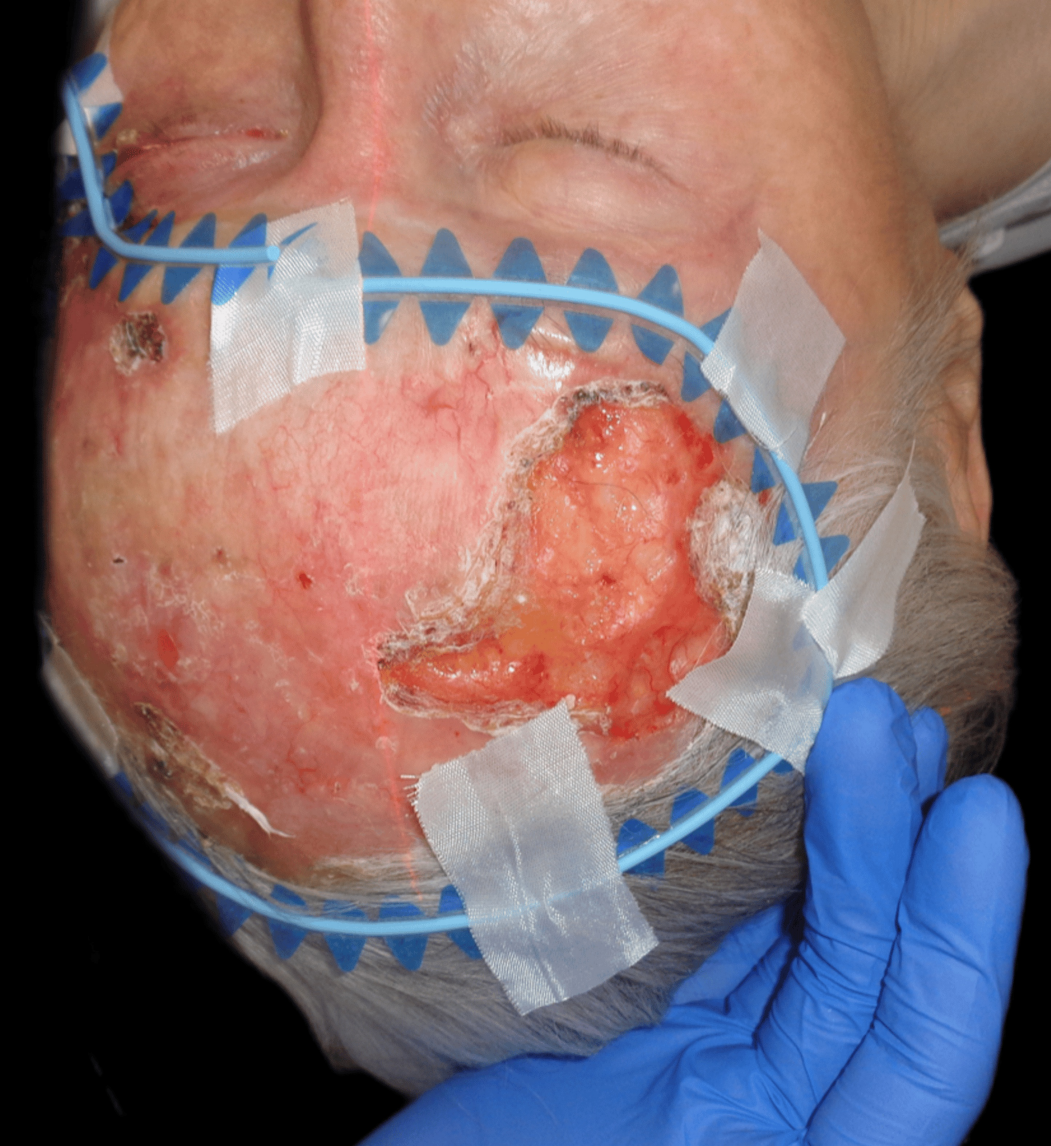
Clinical results after 10 sessions of radiotherapy.

**Figure 11:**
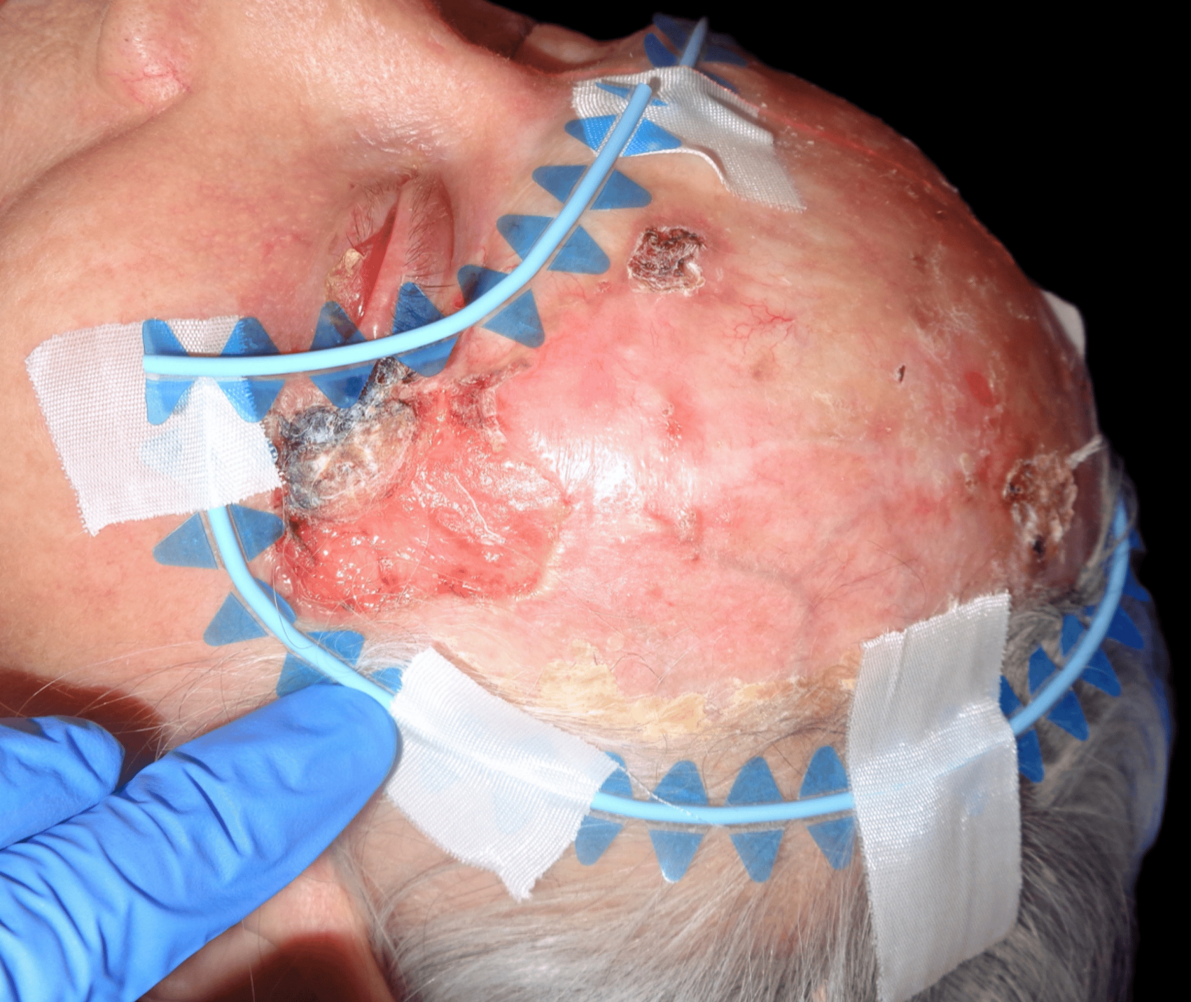
Clinical results after 10 sessions of radiotherapy.

A volumetric modulated arc therapy (VMAT) technique was used, delivering a dose of 70 Gy in 35 sessions, each session delivering 2 Gy, five days per week, for seven weeks. The patient showed a good clinical evolution during the protocol (Figures 10, 11). 

**Figure 12:**
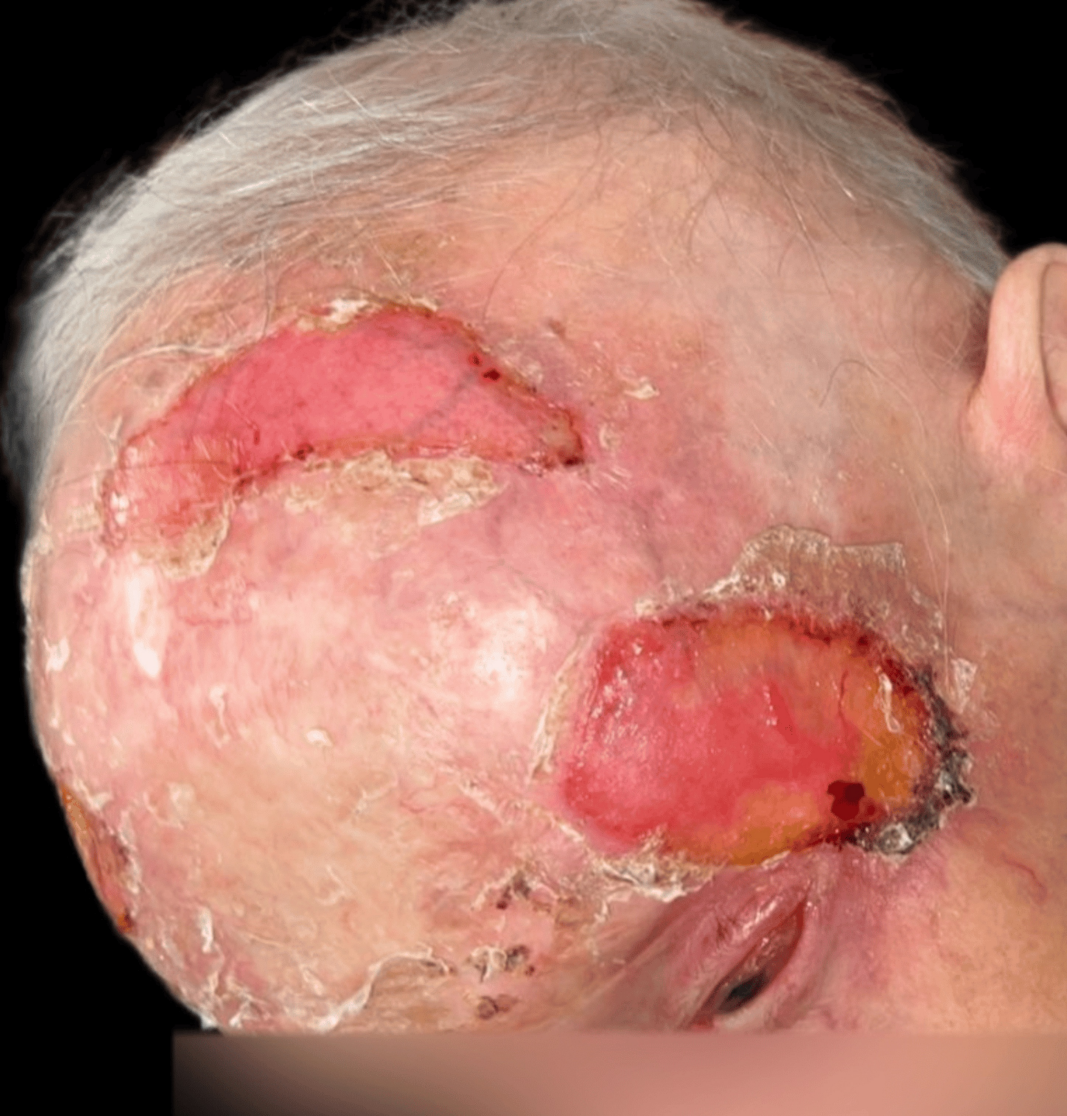
Clinical results at the end of 35 sessions of radiotherapy (last follow-up of the patient on August 1, 2023).

**Figure 13:**
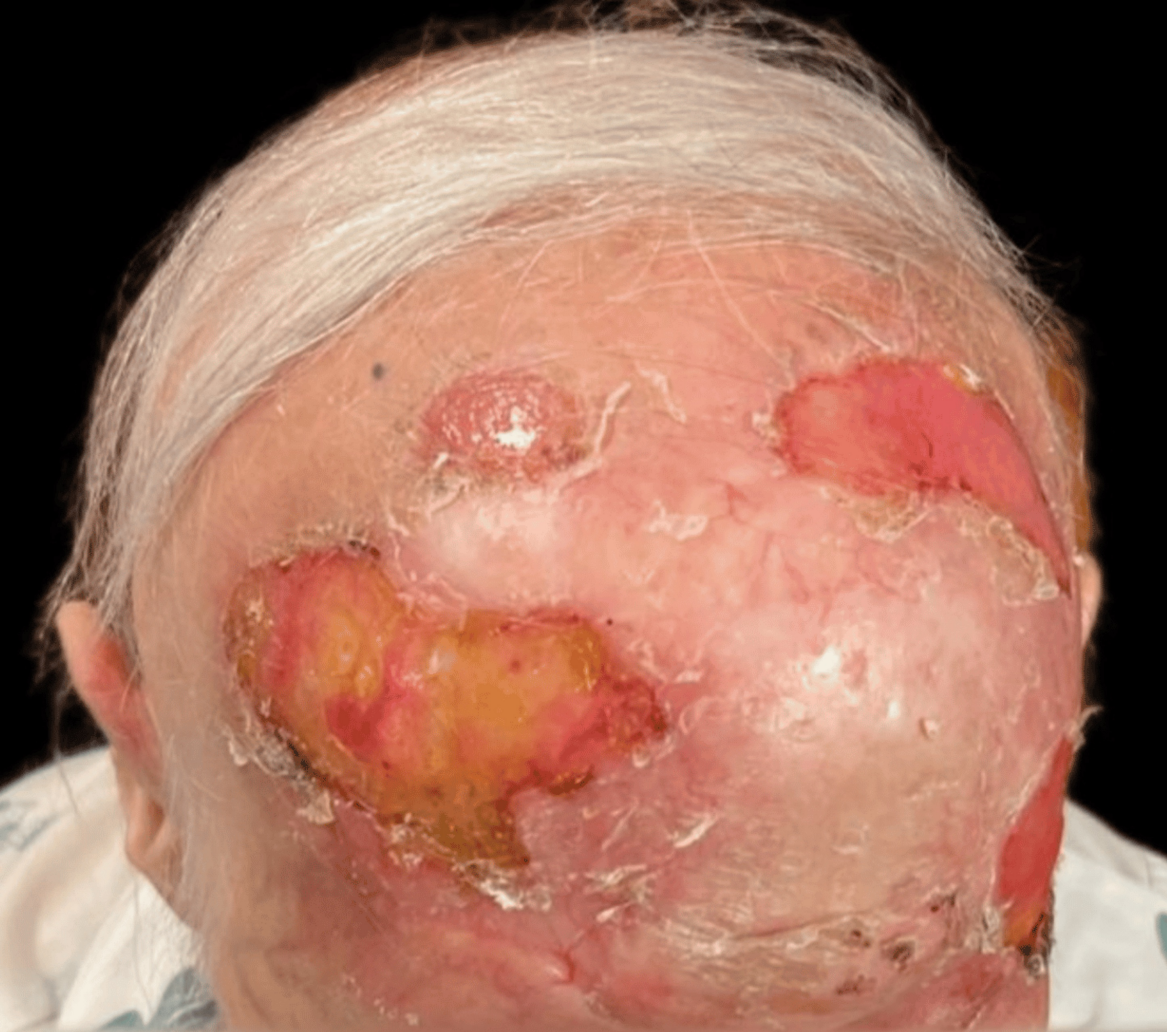
Clinical results at the end of 35 sessions of radiotherapy (last follow-up of the patient on August 1, 2023).

**Figure 14:**
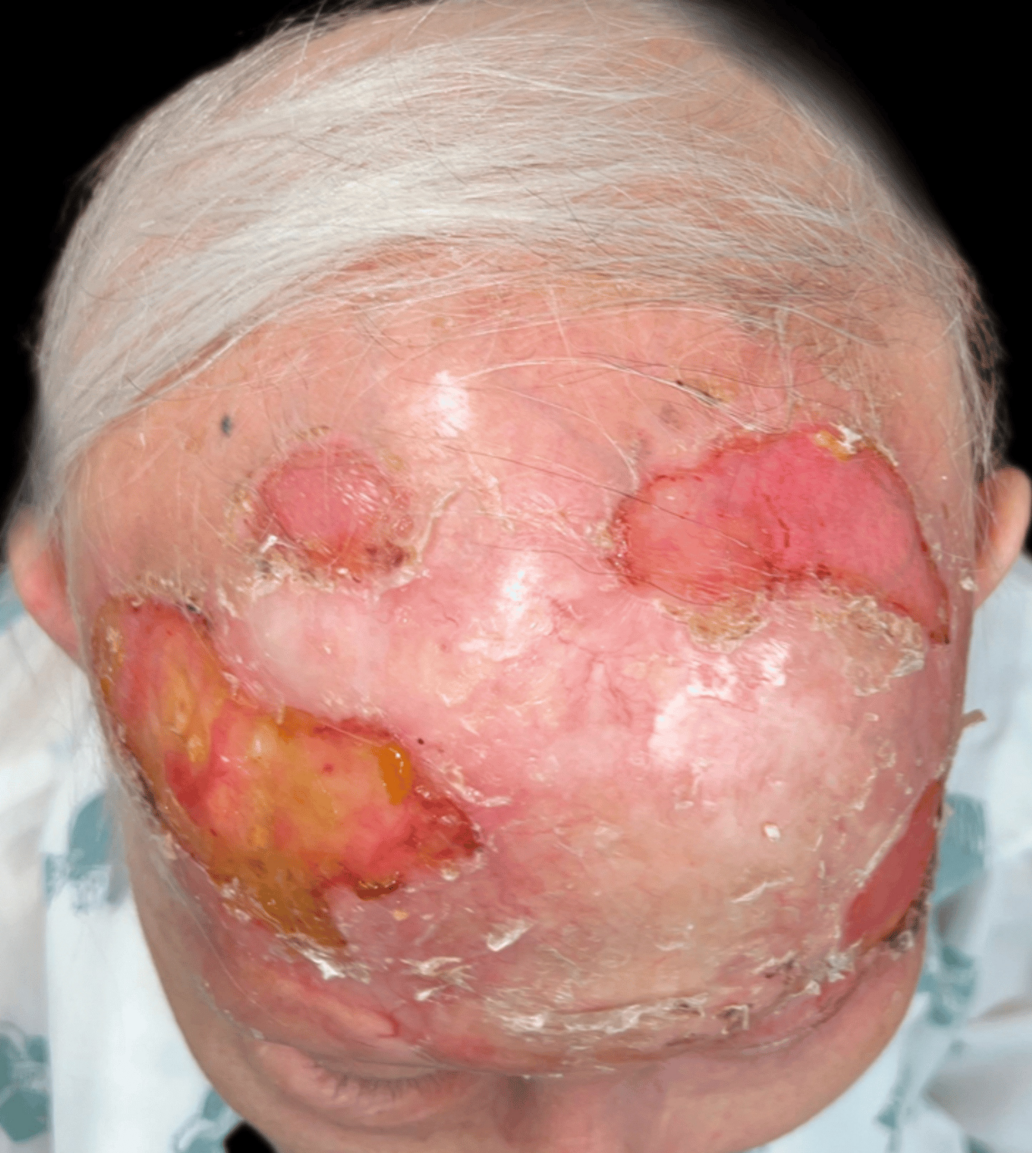
Clinical results at the end of 35 sessions of radiotherapy (last follow-up of the patient on August 1, 2023).

**Figure 15:**
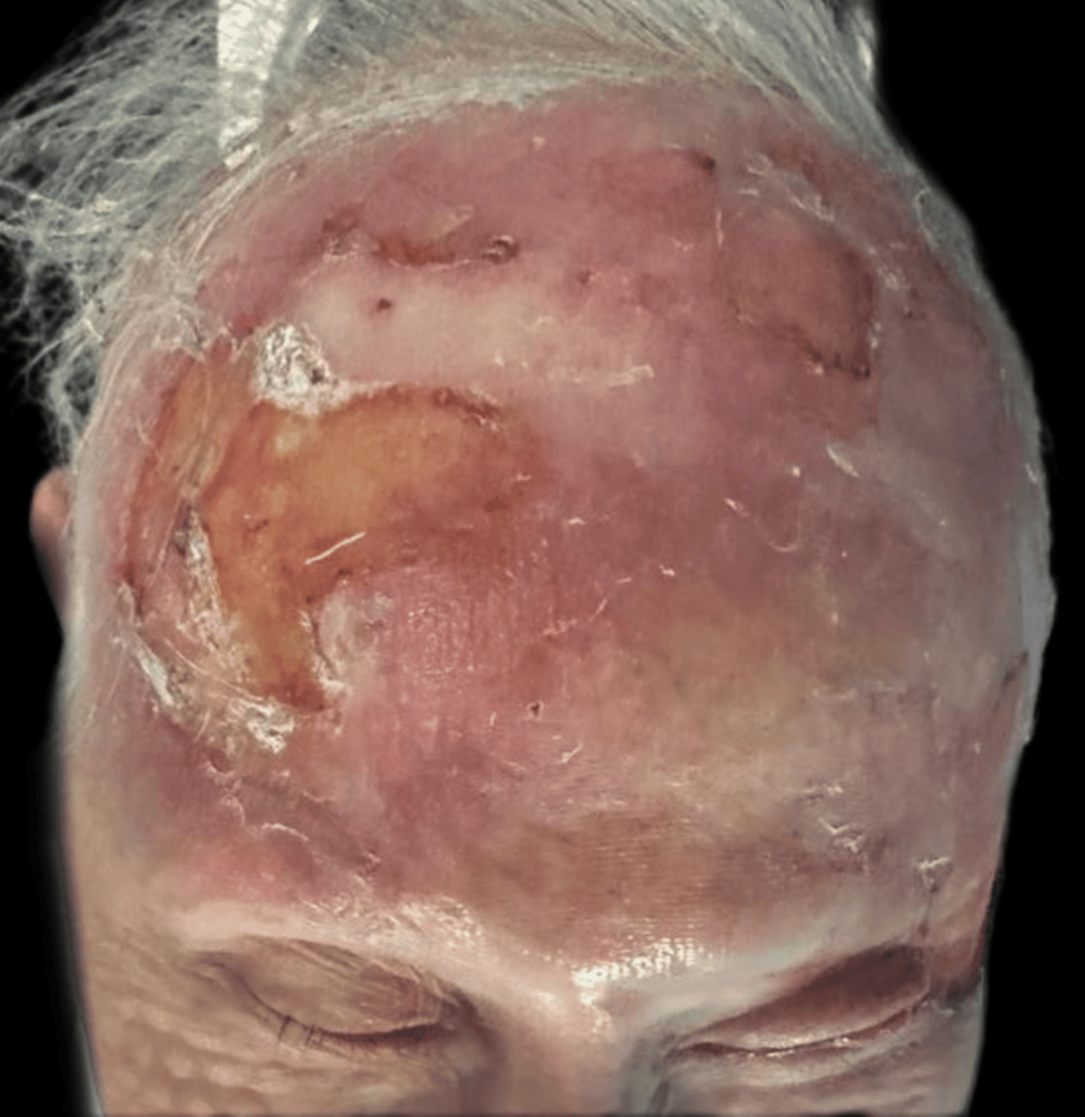
Clinical results at the end of 35 sessions of radiotherapy (last follow-up of the patient on August 1, 2023).

The patient tolerated the treatment well, experiencing mild radiodermatitis that was managed topically. A satisfactory therapeutic response was observed, with clinical and radiological evaluations demonstrating substantial regression in the size of the lesion, epithelialisation of the ulcerations, preservation of eyesight, and no significant toxicities. As the patient herself stated, her quality of life significantly improved following the treatment. The last follow-up was on August 1, 2023, after completing the radiotherapy protocol, with clinical results shown (Figure 12-15).

## Discussion

We present a case with multiple particularities: a large tumour, giant BCC (>5 cm), affecting the scalp (bitemporal, frontal, vertex), of infiltrative histological subtype, with a double recurrence after wide excision and skin grafting, invading the left periorbital area, in an elderly patient who refused the surgical therapeutic approach and could not be treated with Hedgehog inhibitors due to the high costs. Unfortunately, one of the main limitations of this case report is the inability to assess long-term response due to loss to follow-up. This unique, complicated case highlights the importance of choosing the appropriate treatment considering various factors related to the tumour, as well as other patient characteristics.

Actual classification of BCCs

"Advanced BCC", though not precisely defined, typically means that (1) there has been a prolonged period of no treatment and/or repeated treatment failures or recurrences, (2) there is significant destruction of tissue in the surrounding anatomical area, and (3) it is now challenging or unattainable to cure the tumour with surgery (unresectable) or radiation therapy.

BCC has been recently classified into "difficult-to-treat" BCC and "easy-to-treat" BCC, which covers 90% of BCCs [1, 5, 6]. All locally progressed and common BCCs that present unique management challenges for any reason are considered difficult-to-treat BCCs. These factors could include: (1) the tumour's size or location (e.g., eyes, nose, lips, and ears) making it technically difficult to maintain function and aesthetics; (2) the tumour's poorly defined borders, which are frequently linked to the morpheic subtype or recurrence; (3) multiple facial recurrences, which frequently require much larger excision; (4) previous radiation therapy; (5) the patient's unwillingness to accept the consequences of surgery; and (6) the patient's comorbidities interfering with surgery. Increasing treatment difficulties and recurrence risk characterise the heterogeneity of difficult-to-treat BCCs.

Five distinct practical patterns are described by the EADO classification: (1) common BCCs that are challenging to treat due to factors related to the tumour (e.g., location requiring technical skills, poorly defined borders, prior recurrence) and/or the patient (general status, comorbidities, resistance to cooperation); (2) BCCs that are challenging to treat due to the quantity of lesions; (3) broad and/or destructive tumours outside of critical areas; (4) broad and/or destructive tumours in critical or functionally important regions (nose, periorificial); and (5) giant and/or deeply invasive tumours involving extracutaneous tissue [5, 6]. The five clinical scenarios resulted in a four-stage categorisation that encompasses the whole range of BCCs, from common, easily treatable tumours to extremely uncommon metastatic cases. The majority of BCCs, which are low risk and easy to cure, are included in EADO stage I. EADO stage II includes BCCs that are difficult to treat due to their quantity (stage IIB) and common BCCs that are difficult to treat for any reason related to the patient or tumour (stage IIA). Large, destructive tumours that are either on (stage IIIB) or outside (stage IIIA) vital regions are included in stage III, as are severely destructive tumours (stage IIIC). Lastly, mBCCs are classified as stage IV [1, 5, 6]. BCC can be classified, concerning the risk of recurrence, into low and high risk. All difficult-to-treat basal cell carcinomas are at high risk of recurrence [7]. In our case, the tumour we described was a locally advanced, difficult-to-treat and high-recurrence-risk BCC, EADO stage IIIB.

Therapeutic options

The treatment of laBCCs must be discussed in a tumour board, through a multidisciplinary perspective. The inability to completely remove the tumour (R0 resection) and the potential surgical morbidity from complete resection are obstacles to the surgical treatment of locally advanced BCC. Surgery plays a role in palliation and in the neoadjuvant approach, after systemic therapy succeeds in reducing tumour size, allowing a downstaging of the surgical procedure in sensitive areas [8]. Regarding our case, once surgery was denied by the patient, curative radiotherapy or systemic therapy was considered. Recent technical advancements, particularly intensity-modulated and image-guided radiation, which offer high-precision treatments with great local control and a low rate of complications, make it possible to suggest radiotherapy with a curative purpose for laBCC [9, 10]. Because of the possible synergistic effect and the ability to adjust the radiation dose based on the clinical response, there is evidence that combining radiotherapy with systemic therapies (such as immunotherapy or Hedgehog inhibitors) as a neoadjuvant treatment or curative approach leads to encouraging results [11].

With radiation therapy alone, a meta-analysis of almost 10,000 patients from 21 studies covering both BCC and cSCC revealed satisfactory aesthetic outcomes and great five-year local control [12]. In smaller lesions, RT had comparable recurrence rates to excision, Mohs surgery, and other therapeutic options, according to a different systematic review of 40 randomised trials in BCC [13]. Regarding the radiation therapy protocol, with the help of imaging techniques (CT, MRI), the radiation oncologist defines the target area with variable margins, the optimal dose, and the suitable number of fractions, considering the size, depth, and the risk of recurrence of the tumour. Although there is a short-term risk of acute dermatitis, erythema, alopecia, and dryness during the first three to four weeks of treatment, these side effects are usually well tolerated and go away two to three weeks after the treatment is finished. Telangiectasias, skin atrophy, hypopigmentation, hair loss, and secondary cancers are long-term possible adverse effects. The location being treated will determine any further possible adverse effects.

Young age, anatomical regions with poor blood circulation or a high risk of repetitive injuries (such as the elbow or knee), and a history of collagenosis are among the relative contraindications to RT. Patients fully recover from acute adverse effects and tolerate radiation therapy well when receiving the right supportive treatment [9].

The FDA and EMA have approved the use of hedgehog pathway inhibitors (vismodegib and sonidegib), which are targeted inhibitors of the oncogenic protein SMO, to treat patients with laBCC who are not candidates for surgery or radiation therapy. While sonidegib is exclusively recommended for metastatic illness in Australia and Switzerland, vismodegib is also approved for metastatic BCC. For vismodegib and sonidegib, the recommended daily oral dosages are 150 and 200 mg, respectively.

The efficacy of these drugs was studied in two pivotal trials, the ERIVANCE study for vismodegib and the BOLT study for sonidegib [4, 14]. The goal of the nonrandomised, open-label ERIVANCE study was to register 150 mg of vismodegib per day [4, 14]. In contrast, the BOLT study was a double-blind, randomised trial that assessed two distinct dosages of sonidegib (200 and 800 mg/day) [14]. The findings of this research were utilised to register 200 mg/day of sonidegib as the dose that demonstrated the optimal balance between safety and efficacy. According to the mRECIST response, the objective response rate for sonidegib was 56.1% in terms of efficacy [4, 14], while for vismodegib, the objective response rate was 47% [4, 14]. The duration of response for sonidegib was 26 months [4, 14]. Although the two medications were not directly compared, the duration of response for vismodegib in the ERIVANCE study was 9.5 months [4, 14].

Nevertheless, the pharmacokinetic characteristics of these two medications differ. Vismodegib’s volume of distribution, which ranges from 16 to 27 L, indicates that it has low tissue penetration and is mostly diluted into plasma [4, 14]. Sonidegib, on the other hand, has a volume of distribution of >9000 L, suggesting widespread diffusion in the tissues and appears to be more lipophilic than vismodegib [4, 14]. The skin concentration of vismodegib was not measured, while the concentration of sonidegib is said to be six times greater in the skin than in plasma [4, 14]. Sonidegib is the only hedgehog inhibitor with an approved alternate-day dosage in terms of treatment protocols [4, 14].

Common class-specific side effects of available hedgehog inhibitors include fatigue, alopecia, weight loss, myalgia, and dysgeusia [15]. Most patients experience these side effects, and 30% discontinue their medication as a result [15]. No treatment-related deaths were reported [4, 14]. Various approaches, such as drug holidays or dose reduction, have been suggested to avoid or control side effects [1, 2].

In our case, external radiotherapy was performed on the patient with good results; hedgehog inhibitor therapy alone or in combination with radiotherapy could have been an option, but the cost of treatment was an impediment.

Advanced periocular BCC

Although orbital invasion is rare in BCC, when it occurs, the cure rate is decreased. Most cases are recurrent tumours of aggressive histological subtypes (infiltrative, micronodular, or basosquamous) and can lead to severe disfigurement, blindness, or death due to potential intracranial invasion [16, 17]. Clinical indicators of orbital invasion include ocular motility limitation, mass effect-induced globe displacement, eyelid immobility, tumour fixation to the orbital rim, sensory abnormalities, and a visible or palpable mass [16, 17]. Radiological tests complete the evaluation and prepare the therapeutic process.

As treatment options, surgery (globe-sparing surgery-wide excision/Mohs surgery, or in difficult cases, orbital exenteration) is the first line of treatment for periocular BCC [18]. Additionally, radiotherapy is utilised as an adjuvant therapy following surgery or as the sole treatment in patients who cannot undergo surgery [18]. While radiotherapy is effective in inoperable cases, recurrence and complications, such as xerophthalmia, retinopathy, optic neuritis, neovascular glaucoma, cataract formation, and blindness, can occur in one-fourth of cases [19]. Another option is systemic therapy with Hedgehog pathway inhibitors, such as vismodegib or sonidegib. In addition to being used alone, vismodegib has been utilised off-label in conjunction with surgery as an adjuvant or neoadjuvant therapy following surgery [17, 18, 20].

## Conclusions

This case highlights the successful use of palliative radiotherapy in a patient with recurrent giant basal cell carcinoma of the upper face with periorbital invasion, EADO stage IIIB, resulting in good clinical outcomes and preservation of the left eyesight. Radiotherapy is emerging as a valuable treatment option for recurrent BCC in difficult anatomic locations, but careful monitoring and rigorous treatment planning are essential to achieve favourable outcomes while minimising side effects. Future research is needed to establish optimal radiation therapy or combined therapeutic protocols for locally advanced periocular BCCs. Although guidelines indicate multiple therapeutic options for BCCs, and locally advanced BCCs in particular, it is essential to individualise treatment and collaborate in multidisciplinary boards (including dermatologists, oncologists, surgeons, and radiation therapists) to achieve successful outcomes in such challenging, real-life cases.
